# Targeting MerTK decreases efferocytosis and increases anti-tumor immune infiltrate in prostate cancer

**DOI:** 10.1007/s12032-023-02153-z

**Published:** 2023-08-29

**Authors:** Kayla V. Myers Chen, Amber E. de Groot, Sabrina A. Mendez, Mikaela M. Mallin, Sarah R. Amend, Kenneth J. Pienta

**Affiliations:** 1 Cancer Ecology Center, The Brady Urological Institute, Johns Hopkins School of Medicine, 600 N. Wolfe St., Marburg Building Room 113, Baltimore, MD 21287 USA; 2 Department of Pharmacology and Molecular Sciences, Johns Hopkins School of Medicine, Baltimore, MD USA; 3 Department of Oncology, Johns Hopkins School of Medicine, Baltimore, MD USA; 4 Department of Chemical and Biomolecular Engineering, Johns Hopkins Whiting School of Engineering, Baltimore, MD USA

**Keywords:** Macrophage, Efferocytosis, MerTK, Prostate cancer

## Abstract

The prostate cancer tumor microenvironment (TME) is comprised of many cell types that can contribute to and influence tumor progression. Some of the most abundant prostate cancer TME cells are macrophages, which can be modeled on a continuous spectrum of M1-like (anti-tumor macrophages) to M2-like (pro-tumor macrophages). A function of M2-like macrophages is efferocytosis, the phagocytosis of apoptotic cells. Based on literature from other models and contexts, efferocytosis further supports the M2-like macrophage phenotype. MerTK is a receptor tyrosine kinase that mediates efferocytosis by binding phosphatidylserine on apoptotic cells. We hypothesize efferocytosis in the prostate cancer TME is a tumor-promoting function of macrophages and that targeting MerTK-mediated efferocytosis will slow prostate cancer growth and promote an anti-tumor immune infiltrate. The aims of this study are to measure efferocytosis of prostate cancer cells by *in vitro* human M1/M2 macrophage models and assess changes in the M2-like, pro-tumor macrophage phenotype following prostate cancer efferocytosis. Additionally, this study aims to demonstrate that targeting MerTK decreases prostate cancer efferocytosis and promotes an anti-tumor immune infiltrate. We have developed methodology using flow cytometry to quantify efferocytosis of human prostate cancer cells using the LNCaP cell line. We observed that M2 macrophages efferocytose the LNCaP cell line more than M1 macrophages. Following efferocytosis of LNCaP cells by M2 human monocyte-derived macrophages (HMDMs), we observed an increase in the M2-like, pro-tumor phenotype by flow cytometry cell surface marker analysis. By qRT-PCR, flow cytometry, and Western blot, we detected greater MerTK expression in M2 than M1 macrophages. Targeting MerTK with antibody Mer590 decreased LNCaP efferocytosis by M2 HMDMs, establishing the role of MerTK in prostate cancer efferocytosis. In the prostate cancer mouse model hi-myc, *Mertk* KO increased anti-tumor immune infiltrate including CD8 T cells. These findings support targeting MerTK-mediated efferocytosis as a novel therapy for prostate cancer.

**Supplementary Information:**

The online version contains supplementary material available at 10.1007/s12032-023-02153-z.

## Introduction

Phagocytosis of apoptotic cells, or efferocytosis, is an essential process for contexts with high apoptotic cell turnover, including embryonic development, lymphocyte development, and resolution of inflammation. Efferocytosis by macrophages has been studied extensively in the field of immunology, focusing on its function in maintaining tissue homeostasis and resolving inflammation [1–3]. Following efferocytosis, phagocytosed lipid components and metabolites bind PPAR and LXR nuclear receptors, inducing transcription of M2-associated genes [4–7]. Additionally, downstream signaling from receptors that bind PtdSer, such PI3K/AKT and STAT3, also polarizes macrophages to an M2-like phenotype [8, 9]. Clearance of apoptotic cells prevents their progression to secondary necrosis, or late apoptosis. Secondary necrotic cells have permeabilized membranes, resulting in release of danger-associated molecular patterns (DAMPs) that induce M1-like macrophage polarization [10–12]. It has also been shown that secondary necrotic cells release STimulator of Interferon Genes (STING) ligand cyclic guanosine monophosphate–adenosine monophosphate (cGAMP) [13]. STING agonists also increase M1-like polarization, a further mechanism by which secondary necrotic cells promote an anti-tumor macrophage phenotype [14, 15]. Altogether, it is hypothesized that efferocytosis is a tumor-promoting function by increasing M2-like polarization and preventing the apoptotic cell from progressing to secondary necrosis and promoting M1-like polarization.

There have been limited studies profiling changes in macrophage phenotype following efferocytosis of cancer cells. Overall, these studies reflect what has been established with non-cancer models: Efferocytosis increases the M2-like pro-tumor phenotype and decreases the M1-like anti-tumor phenotype. Efferocytosis of the breast cancer cell line MCF-7 by the mouse macrophage cell line Raw267.4 increases the expression of M2-associated cytokines IL-4 and IL-10, and expression of *Tgfb1* mRNA [16]. In THP-1 macrophages, efferocytosis of breast cancer cell lines MDA-MB-231 and 4T1 increases IL-10 and decreases IL-6 secretion, an M1-associated cytokine. Efferocytosis of mouse colon cancer cell line SL4 increased expression of *Arg1*, *Tgfb1* and *Il10* mRNA and CD206 protein in mouse bone marrow-derived macrophages [8]. In prostate cancer, efferocytosis of mouse cell line RM-1 by bone marrow-derived macrophages increased CD206 protein and *Il10*, *Tgfb1*, *Chi3l3,* and *Arg1* mRNA [9]. Efferocytosis of prostate cancer cells by mouse bone marrow-derived macrophages induced a pro-inflammatory cytokine secretion profile, a unique phenotype change following efferocytosis in this context [17]. Despite efferocytosis being an emerging interest in cancer biology, it remains unknown how efferocytosis of human prostate cancer cells by human macrophages alters phenotype.

Tyro3, Axl, and MerTK (TAM receptors) are a family of receptor tyrosine kinases that mediate efferocytosis by macrophages through binding phosphatidylserine on apoptotic cells. Inhibiting the TAM receptors has been shown to block efferocytosis by macrophages and other cell types and serve as promising targets for blocking efferocytosis in tumors [18, 19]. The majority of TAM receptor-mediated efferocytosis studies with both cancer and non-cancer models focus on MerTK. A MerTK targeting antibody decreased mouse thymocyte efferocytosis *in vitro* and *in vivo* [13]. In murine primary mammary epithelial cells, *Mertk* knockout decreased efferocytosis of the human T cell lymphoblast like cell line CEM [20]. Additionally, *Mertk* knockout in primary mouse macrophages decreases efferocytosis of cardiomyocytes [21]. Mertk knockdown and MerTK inhibition with small molecule UNC2025 decreased efferocytosis of K7M2 cells by mouse bone marrow-derived macrophages and macrophage cell line RAW264.7 [22]. Additionally, in a lung adenocarcinoma mouse model, MerTK inhibition in combination with anti-PD1 and radiotherapy has been shown to decrease tumor growth and lead to an abscopal effect [24]. These studies support macrophage MerTK as an emerging target for anti-cancer therapy, but the role of MerTK in prostate cancer cell efferocytosis remains unknown. Additionally, MerTK expression on prostate cancer tumor-associated macrophages is unknown, although MerTK expression in prostate cancer cell lines and mouse models has been investigated [23]. In this study, we confirm the expression of MerTK in our human macrophage models. We investigate targeting MerTK to decrease efferocytosis of prostate cancer cells *in vitro*. Finally, we investigate if *Mertk* KO promotes an anti-tumor immune infiltrate in vivo utilizing the hi-myc prostate cancer genetically engineered mouse model.

## Materials and methods

### Human monocyte-derived macrophage (HMDM) culture

Human monocytes were isolated and M1 and M2 macrophages were generated using previously published methods [24]. In brief, human PBMCs were acquired from the New York Blood Center (New York, NY) from healthy donors aged between 20 and 40. PBMCs were processed with a PBS wash and red blood cell lysis with ACK lysing buffer (118-156-101, Quality Biological). CD14 + monocytes were isolated by magnetic bead separation (17,858, STEMCELL Technologies) and cryopreserved in 95% heat-inactivated fetal bovine serum (HI-FBS) (16,140,071, Gibco) and 5% DMSO (4-X, ATCC).

Monocytes were thawed in RPMI (11,875,119, Gibco) supplemented with 10% HI-FBS and 1% penicillin–streptomycin (11,995,073, Gibco). For M1 macrophages, monocytes were differentiated with 20 ng/mL granulocyte macrophage colony-stimulating factor (GM-CSF) (300-03, PeproTech) for five days, followed by M1 polarization with 20 ng/mL GM-CSF, IFNγ (300–02, PeproTech), LPS (L3012, Sigma-Aldrich), and IL-6 (200-06, PeproTech) for four days. M2 macrophages were differentiated with 20 ng/mL macrophage colony-stimulating factor (M-CSF) (300-25, PeproTech) for five days, followed by M2 polarization with 20 ng/mL M-CSF, IL-4 (200-04, Peprotech), IL-13 (200–13, Peprotech), and IL-6.

### THP-1 culture, macrophage differentiation, and polarization

THP-1 (TIB-202, ATCC) cells were cultured in RPMI supplemented with 10% HI-FBS and 1% penicillin–streptomycin and maintained at a confluency of 0.1 × 10^6^–1 × 10^6^ cells/mL. THP-1 cells were authenticated and tested for *mycoplasma* biannually (Genetica). THP-1 cells were differentiated into macrophages by 100 nM PMA (1,652,981, BioGems) for one day, followed by one-day recovery in fresh media without PMA. For M1 polarization, 20 ng/mL GM-CSF was supplemented during differentiation, followed by three days of incubation with 20 ng/mL GM-CSF, IFNγ, LPS, and IL-6. For M2 polarization, 20 ng/mL M-CSF was supplemented during differentiation, followed by three days of incubation with 20 ng/mL M-CSF, IL-4, IL-13, and IL-6.

### Cell culture and induction of apoptosis

The prostate cancer cell line LNCaP (CRL-1740, ATCC) was maintained in RPMI supplemented with 10% FBS (97,068–085, Avantor) and 1% penicillin–streptomycin. Apoptotic LNCaP cells for efferocytosis assays were prepared by treating with 120 μM cisplatin (232,120, Millipore Sigma) during plating. After 24 h of incubation, remaining non-adherent cells were collected and washed twice with PBS to remove cisplatin prior to the addition to macrophages. Cells were determined to be apoptotic by Annexin V staining. Cells were resuspended in Annexin V binding buffer (422,201, BioLegend) and stained with FITC-Annexin V (640,906, BioLegend) and 7-AAD (420,403, BioLegend) for 10 min at room temperature. Data were collected using a Bio-Rad S3e Cell Sorter and analysis was performed using FlowJo. Quadrant gating was defined using unstained and single-stain controls. Cells were approximately 16% live (Annexin V^−^/7-AAD^−^), 55% early apoptotic (Annexin V^+^/7-AAD^−^), and 30% late apoptotic (Annexin V^+^/7-AAD^+^) (Supplementary Fig. 1).

### Prostate cancer efferocytosis by M1 and M2 human macrophages

HMDMs were labeled on Day 5 prior to cytokine addition, and THP-1 cells were labeled on Day 0 prior to differentiation with PMA with CellTrace Yellow (C34567, Invitrogen) or CellTrace Far Red (C34564, Invitrogen) at 1:1000 in PBS for 30 min. LNCaP cells were labeled with CellTrace CFSE (C34554, Invitrogen) or CellTrace Violet (C34557, Invitrogen) at 1:1000 in PBS prior to apoptosis induction with cisplatin. Apoptotic LNCaP was added at a 5:1 apoptotic LNCaP cell:macrophage ratio in fresh media without cytokines. After 24 h, cells were harvested using trypLE Express (12,604–013, Gibco) and scraping. Cells were washed in PBS and resuspended in FACS buffer (PBS with 5% BSA and 2 mM EDTA). Efferocytosis quadrant gating was defined using a CellTrace Yellow- or CellTrace Far Red-labeled macrophage, unlabeled LNCaP cell control. Data were collected using an Attune NxT flow cytometer (Thermo Fisher Scientific) and analysis was performed using Kaluza (Beckman Coulter).

### Macrophage cell surface marker flow cytometry analysis

M1 and M2 HMDMs and THP-1 macrophages were dissociated using enzyme-free cell dissociation buffer (13,151,014, Gibco) and scraping. Macrophages were stained with cell surface marker antibody or corresponding isotype control listed in Supplementary Table 1. Cells were resuspended in FACS buffer with 7-AAD viability dye (420,404, BioLegend) and incubated for 10 min at room temperature prior to data acquisition. Data were collected using an Attune NxT flow cytometer or Bio-Rad S3e flow cytometer and analysis was performed using Kaluza or FlowJo. Live macrophages were gated as 7-AAD negative cells. Delta median fluorescence intensity (MFI) was calculated as MFI of positive stained cells minus MFI of the isotype control stained cells.

### TAM receptor gene expression

Expression levels of TAM receptor mRNA were assessed by the human nCounter Myeloid Innate Immunity Panel (*MERTK*, Accession Number: NM_006343.2) and Custom Panel Plus (*AXL*, Accession Number: NM_021913 and *TYRO3*, Accession Number: NM_006293) (NanoString Technologies) from our previously published study [25]. Hybridization of samples was performed using 75–100 ng of RNA measured by Nanodrop 2000 Spectrophotometer (Thermo Scientific). Gene expression was analyzed with nSolver Software 4.0 (NanoString Technologies). The expression levels of each gene were normalized to those of the control genes.

For qRT-PCR, cells were dissociated with trypLE Express (12,604–013, Gibco) with scraping. RNA was extracted with RNeasy Mini kit (74,104, Qiagen). RNA purity and concentration were measured by Nanodrop 2000 Spectrophotometer (Thermo Scientific). RNA was converted to complementary DNA (cDNA) with iScript cDNA synthesis kit (1,708,890, Bio-Rad). qRT-PCR was performed with SsoFast EvaGreen Supermix (1,725,201, Bio-Rad) in technical replicates. *UBC* was selected as a housekeeping gene for HMDM Biological Replicate 1 and THP-1 macrophages. *RPL13A* was selected as a housekeeping gene for HMDM Biological Replicate 2. Gene expression was normalized to the housekeeping gene and calculated with the delta-delta Ct method [26]. TAM receptor primer sequences were obtained through PrimerBank (https://pga.mgh.harvard.edu/primerbank/index.html). Stable housekeeping genes for macrophage studies and primer sequences were selected from the publication by Kalagara et al. [27] Primer sequences are listed in Supplementary Table 2.

### Western Blot analysis

Cells were collected in cold PBS supplemented with Halt Protease and Phosphatase Inhibitor Cocktail (78,442, ThermoFisher) and spun down. Pellets were lysed in RIPA buffer (R0278, Sigma) supplemented with Halt Protease and Phosphatase Inhibitor Cocktail. Lysates were incubated on a rotator at 4 °C for 30 min, then spun down at maximum speed for 10 min. Supernatant was stored at − 80 °C. Protein concentration was determined using a BCA assay (23,225, ThermoFisher Scientific) and 50 μg protein was prepared for gel electrophoresis with 4 × Laemmli Sample Buffer (161–047, Bio-Rad) supplemented with 2-β-mercaptoethanol (161–0710, Bio-Rad). Samples were boiled at 99 °C, run on a 4–20% SDS-PAGE gel (4,561,094, Bio-Rad) and transferred onto a nitrocellulose membrane (1,704,158, Bio-Rad). Membranes were blocked with Casein Blocking Buffer (B6429, Sigma-Aldrich) and incubated with primary antibodies for MerTK (3419, Cell Signaling Technologies, diluted 1:1000 in Casein), pMerTK (Y749 + Y753 + Y754) (ab14921, Abcam, diluted 1:750 in Casein) and β-actin (A5441, Sigma-Aldrich, diluted 1:5000 in Casein) overnight at 4 °C under agitation. Membranes were incubated with anti-rabbit (926–32,211, LI-COR, diluted 1:15,000 in Casein) and anti-mouse (926–69,070, LI-COR, diluted 1:20,000 in Casein) secondary antibodies and imaged on an Odyssey (LI-COR). pMerTK and MerTK signal were quantified using ImageJ. Relative Intensity is defined mean pixel intensity of pMerTK (normalized to β-actin) divided by the mean pixel intensity of MerTK (normalized to β-actin) with relative intensity of M2 Ctrl set to 1.

### MerTK inhibition in human macrophages

Mer590 (MABS2246, Millipore Sigma) and mouse IgG1 isotype control (MAB002, R&D systems) dilutions were prepared in fresh media. THP-1 M2 macrophages were incubated in media containing antibody for two hours prior to collection for MerTK Western Blot analysis. CellTrace Far Red-labeled M2 HMDMs were incubated with media containing antibody for two hours prior to CellTrace Violet-labeled apoptotic LNCaP addition. After a 6-h of coculture, macrophages were collected for efferocytosis assays as described.

### Generation of Mertk KO, hi-myc mouse model

The Johns Hopkins Institutional Animal Care and Use Committee approved all experiments involving mice (protocol numbers MO19M41 and MO21M471). FVB/N hi-Myc was a gift from Brian Simons (Baylor University). C57BL6/J *Mertk* KO was a gift from Greg Lemke (Salk Institute). FVB/N *Mertk* KO, hi-myc mice were generated by crossing C57BL6/J *Mertk* KO with FVB/N hi-myc mice six times. After crosses two through five, tail snips were analyzed for 100 single-nucleotide polymorphisms (SNPs) between C57BL6 and FVB mice (Laboratory Animal Genetics Services, University of Texas MD Anderson Cancer Center) and mice with the highest percentage of FVB SNPs were selected for the next round of breeding. FVB/N *Mertk* KO, hi-myc mice are estimated to have a greater than 99.5% FVB background. Male hi-myc transgenic mice of both *Mertk* WT and *Mertk* KO genotypes were aged to 2, 6, and 12 months for analysis. Tumor tissue fixation and histology imaging were performed as previously described [28]. Representative histology images of *Mertk* WT and KO tumors aged to 2, 6, and 12 months are shown in Supplementary Fig. 2.

### Mouse prostate flow cytometry immune cell analysis

Ventral (V), dorsal (D), and lateral (L) mouse prostate lobes were dissected, combined, and subjected to single-cell dissociation using the MACS Mouse Tumor Dissociation Kit (Miltenyi 130–096-730) and gentleMACS Dissociator (Miltenyi). Suspended cells were blocked with rat serum (012–000-120, Jackson ImmunoResearch), stained with FVS570 viability dye (1 μl/mL, 564,995, BD Biosciences) or LIVE/DEAD Fixable Yellow (1 μl/mL, L34959, Thermo Fisher Scientific) in the dark for 15 min at room temperature. Samples were washed with PBS and incubated with myeloid extracellular antibody panel (Supplementary Table 3), lymphocyte extracellular antibody panel (Supplementary Table 4), or corresponding isotype panels diluted in Brilliant Stain Buffer (566,349, BD Biosciences) in the dark for 30 min at 4 degrees Celsius. Cells were washed with FACS buffer, fixed with 1 × Fixation Buffer (420,801, BioLegend) in the dark for 20 min at room temperature, and stored overnight in FACS buffer at 4 degrees Celsius. Samples were incubated in 1 × FoxP3 Fix/Perm Solution (421,401, BioLegend) in the dark for 20 min at room temperature and washed with 1 × FoxP3 Perm Buffer (421,402, BioLegend). Cells were resuspended with myeloid intracellular antibody panel (Supplementary Table 3) or corresponding isotype panels diluted in FACS buffer in the dark for 30 min at room temperature under gentle agitation. Cell suspensions were washed with FACS buffer and analyzed with an Attune NxT flow cytometer and analysis was performed with Kaluza Analysis Software. Immune cell population markers are defined in Supplementary Table 5.

### Statistical analysis

Data were analyzed using t-test via GraphPad Prism. Differences were considered significant at *p* < 0.05. Figures denote statistical significance of ns as not significant, *p* < 0.05 as *, *p* < 0.01 as **, *p* < 0.001 as *** and *p* < 0.0001 as ****.

## Results

### M2 HMDMs and THP-1 macrophages efferocytose LNCaP cells at higher levels than M1s

**Fig. 1:**
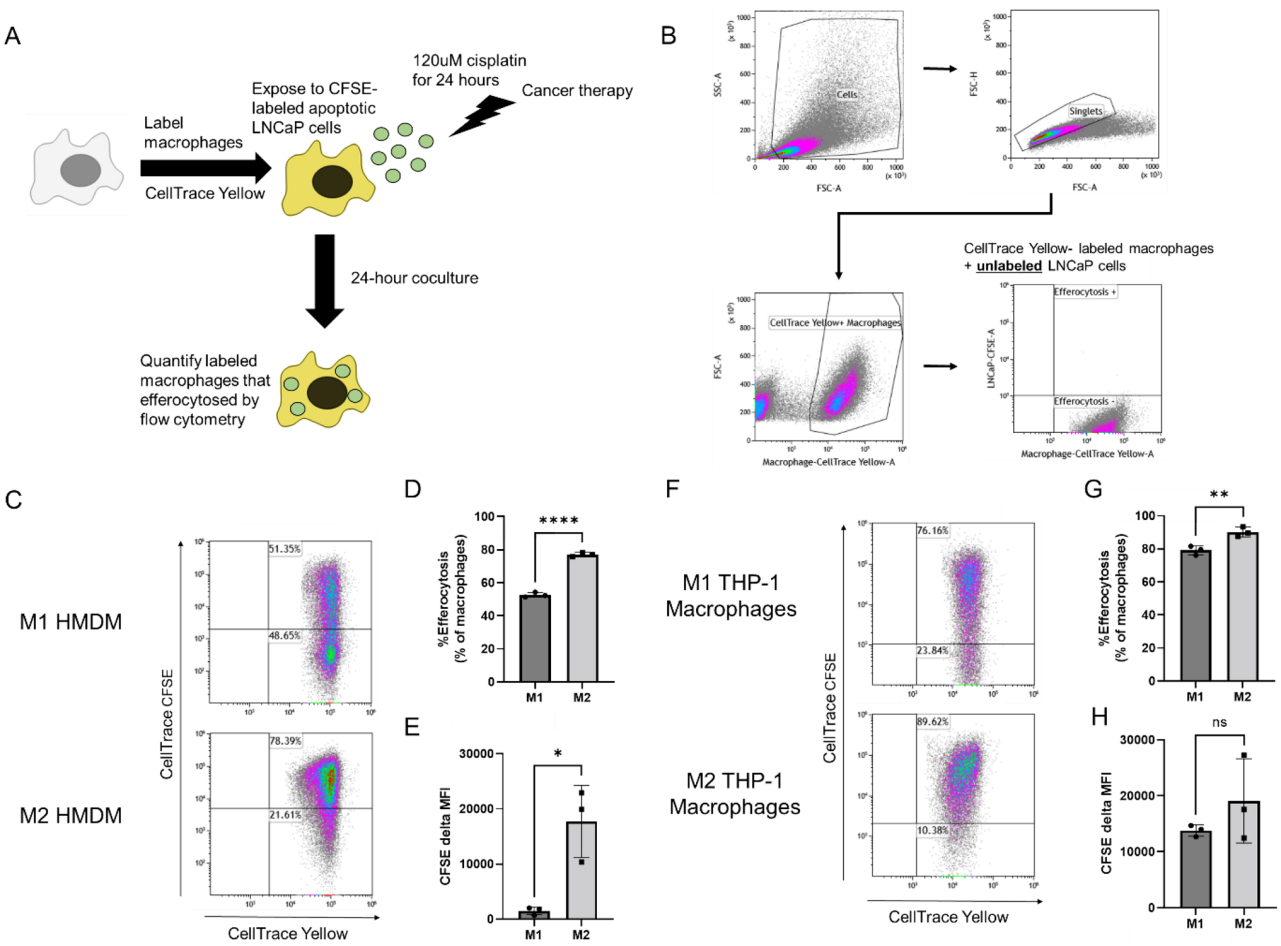
M2 human macrophages efferocytose LNCaP cells at higher levels than M1s. Experimental scheme for measuring efferocytosis of prostate cancer cells (**A**). Efferocytosis gating strategy using CellTrace Yellow-labeled macrophages and unlabeled LNCaP cell control (**B**). Efferocytosis of LNCaP cells by M1 and M2 HMDMs (**C**). Quantified %efferocytosis (**D**) and CFSE delta MFI (**E**) by M1 and M2 HMDMs. Efferocytosis of LNCaP cells by M1 and M2 THP-1 macrophages (**F**). Quantified %efferocytosis (**G**) and CFSE delta MFI (**H**) by M1 and M2 THP-1 macrophages. CFSE delta MFI was calculated as MFI of macrophages + CFSE LNCaP minus MFI of macrophages + unlabeled LNCaP. Significance was calculated by t-test with * *p* < 0.05, **p < 0.01 and **** *p* < 0.0001

To investigate the efferocytosis levels of human prostate cancer cells between M1 and M2 human macrophages, efferocytosis assays were performed with HMDMs and THP-1 macrophages (Fig. 1A). To account for differences in autofluorescence between samples, efferocytosis gating was defined using a CellTrace Yellow-labeled macrophage and unlabeled LNCaP cell coculture control (Fig. 1B). Across three biological replicates, M2 HMDMs displayed greater efferocytosis of LNCaP cells than M1 HMDMs (Fig. 1C). This was quantified as efferocytosing macrophages as a percentage of total macrophages (Fig. 1D) and CFSE delta MFI (Fig. 1E). Similarly, M2 THP-1 macrophages efferocytosed LNCaP cells at higher levels than M1 THP-1 macrophages (Fig. 1F). This was quantified as efferocytosing macrophages as a percentage of total macrophages (Fig. 1G). There was no significant difference in CFSE delta MFI between M1 and M2 THP-1 macrophages (Fig. 1H).

### LNCaP efferocytosis increases pro-tumor cell surface markers CD206 and PDL1

**Fig. 2:**
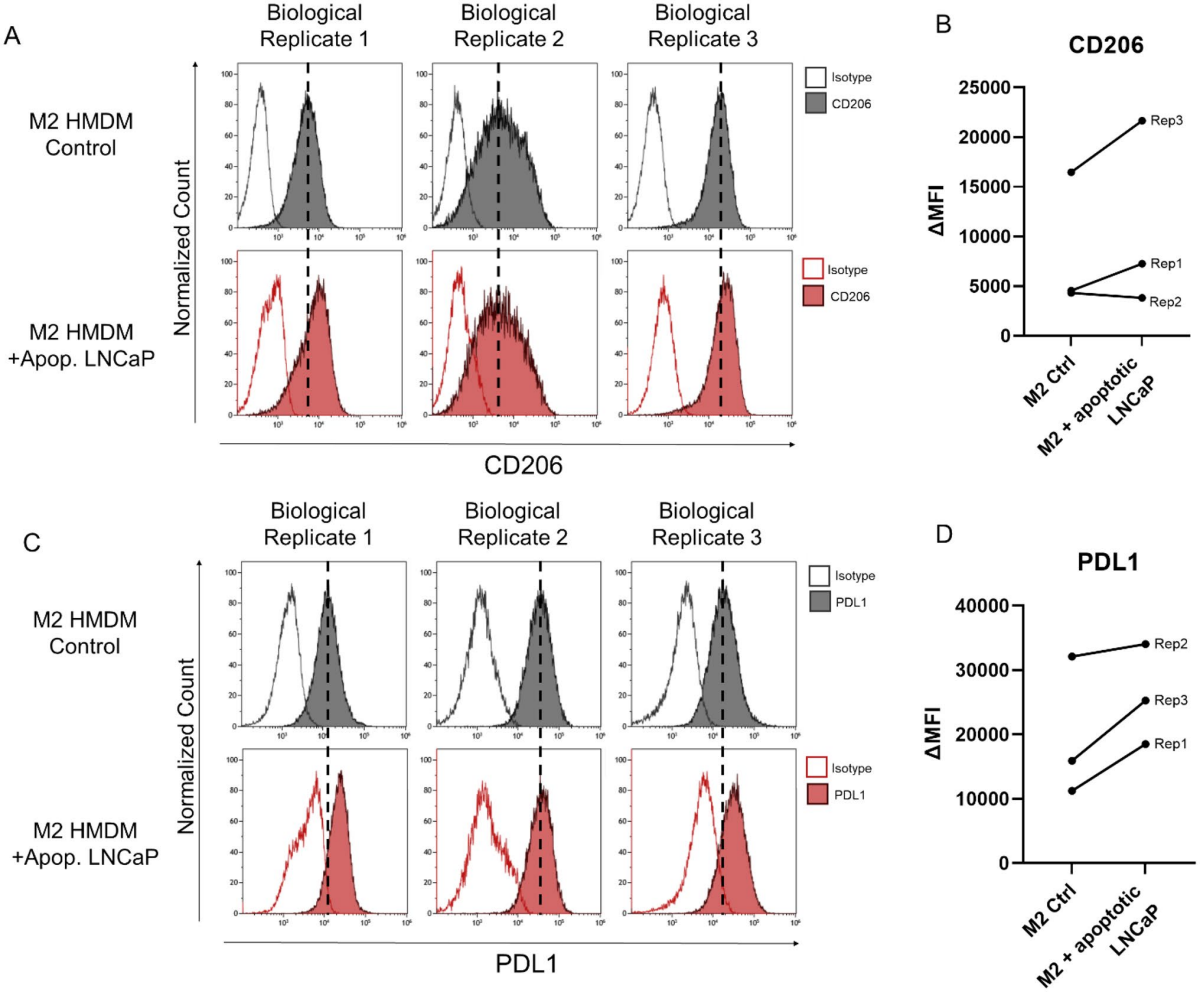
LNCaP efferocytosis increases the pro-tumor macrophage phenotype in M2 HMDMs. CD206 expression (solid) and isotype control (outline) in three biological replicates of control M2 HMDMs (gray) and M2 HMDMs incubated with apoptotic LNCaP cells for 24 h (red) (**A**). PDL1 expression (solid) and isotype control (outline) in three biological replicates of control M2 HMDMs (gray) and M2 HMDMs incubated with apoptotic LNCaP cells for 24 h (red) (**C**). Δ median fluorescence intensity (ΔMFI) for CD206 (**B**) and PDL1 (**D**) is calculated as MFI of positive stained minus MFI of isotype control stained. Dashed lines indicate the MFI of the positive-stained M2 HMDM control sample

Following LNCaP cell efferocytosis, we profiled changes in pro-tumor cell surface markers CD206 and PDL1 in three biological replicates of M2 HMDMs incubated with apoptotic LNCaP cells. Incubation with apoptotic LNCaP cells increased the expression of CD206 in Biological Replicate 1 and 3 and decreased the expression of CD206 in Biological Replicate 2 (Fig. 2A, B). Apoptotic LNCaP cell incubation increased expression of PDL1 across all three biological replicates (Fig. 2C, D).

### MerTK is expressed higher in M2 than M1 human macrophages

**Fig. 3:**
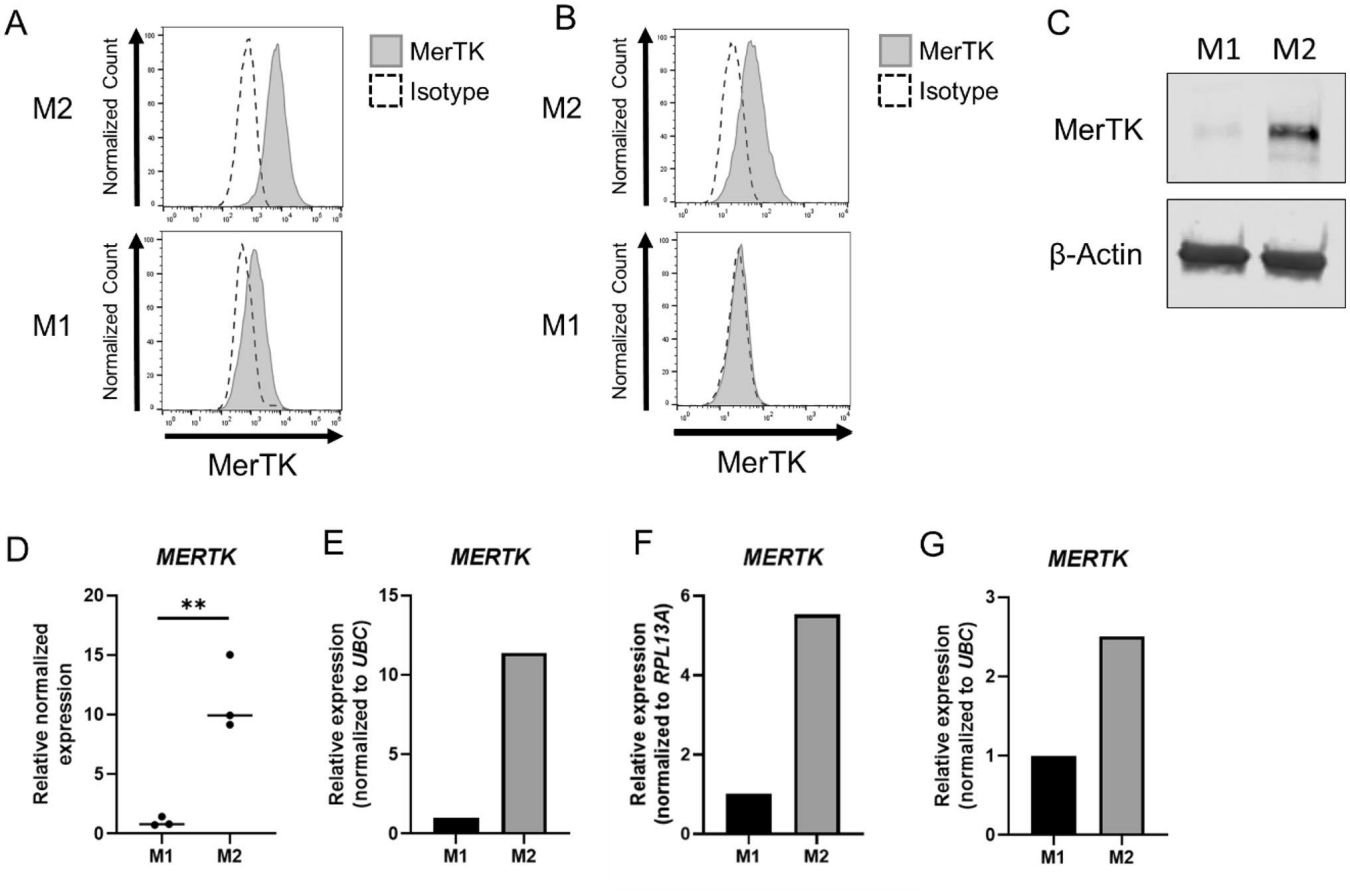
MerTK is expressed higher on M2 than M1 human macrophages. Flow cytometry analysis of MerTK on M1 and M2 HMDMs (**A**) and THP-1 macrophages (**B**). Western blot analysis of MerTK in M1 and M2 HMDMs with β-actin as a loading control (**C**). *MERTK* mRNA expression by NanoString analysis in three biological replicates of M1 and M2 HMDMs (**D**). *MERTK* mRNA expression by qRT-PCR in two biological replicates of M1 and M2 HMDMs (**E**, **F**) and M1 and M2 THP-1 macrophages (**G**). Flow cytometry and Western blot data in HMDMs are representative of three biological replicates. **p < 0.01

MerTK protein was expressed higher in M2 HMDMs than M1 HMDMs by flow cytometry and Western Blot in three biological replicates (Fig. 3A, C). MerTK protein is also expressed higher in M2 than in M1 THP-1 macrophages by flow cytometry (Fig. 3B). At the mRNA level, in HMDMs, *MERTK* is increased in M2s compared to M1s in three biological replicates by a NanoString Panel and two biological replicates by qRT-PCR (Fig. 3D-F). M2 THP-1 macrophages also have elevated *MERTK* mRNA compared to M1 THP-1 macrophages (Fig. 3G).

### Targeting MerTK decreases LNCaP efferocytosis

**Fig. 4:**
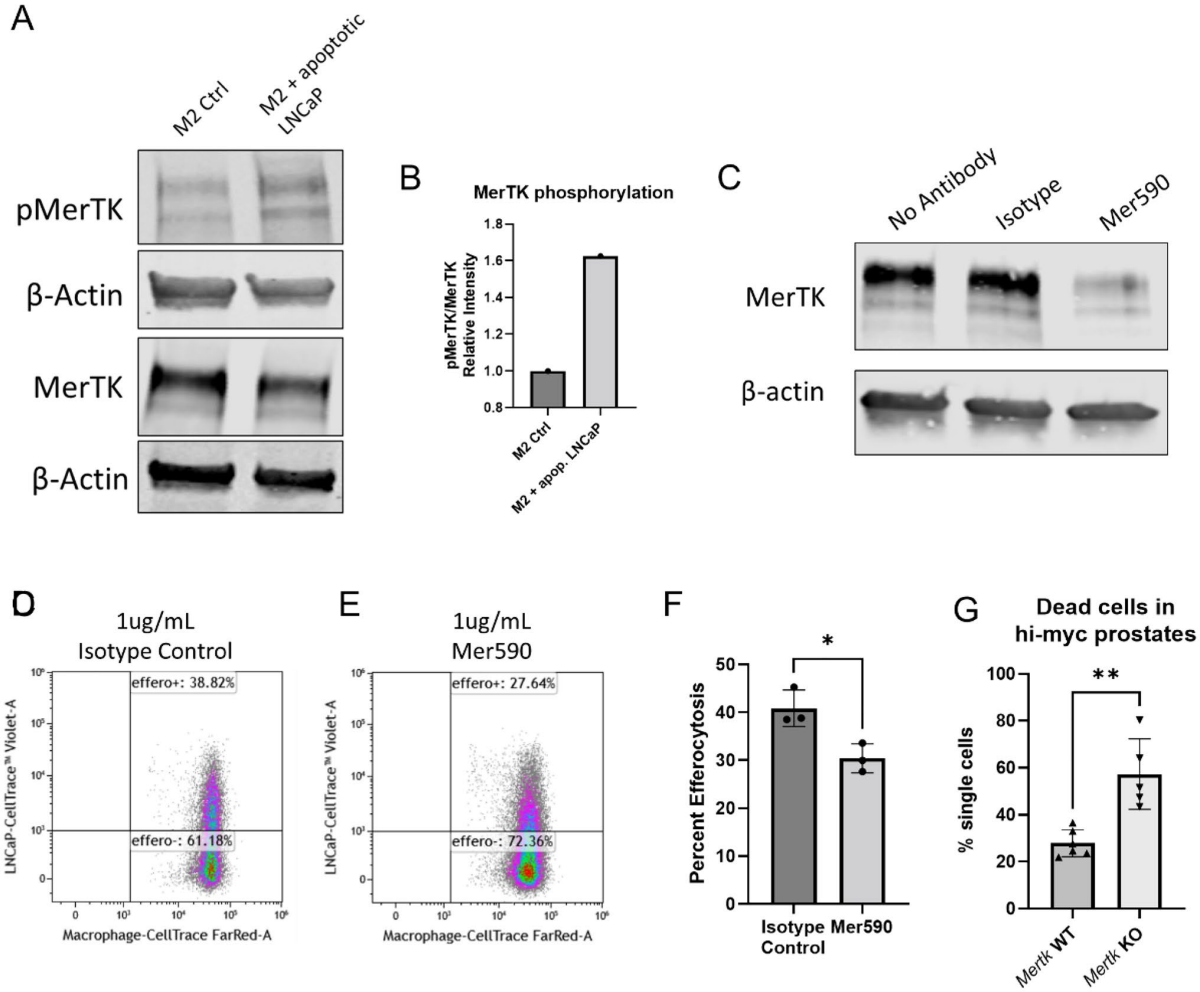
Targeting MerTK decreases prostate cancer efferocytosis. Phosphorylated MerTK (pMerTK) Western blot analysis in M2 THP-1 macrophages and M2 THP-1 macrophages incubated with apoptotic LNCaP cells for 1 h (**A**). Quantification of the ratio of relative intensity of pMerTK signal to MerTK signal (**B**). MerTK Western blot analysis in M2 HMDMs treated with isotype control of Mer590 for two hours (**C**). Representative examples of 6-h efferocytosis flow cytometry assays with M2 HMDMs pre-incubated with isotype control antibody (**D**) or Mer590 (**E**) and percent efferocytosis quantified in three biological replicates (**F**). Percent dead cells in 2-month hi-myc *Mertk* WT and KO prostates as percentage of LIVE/DEAD fixable yellow positive single cells (**G**). * *p* < 0.05, ** *p* < 0.01

Phosphorylated MerTK (pMerTK) increases 1 h following apoptotic LNCaP cell addition in M2 THP-1 macrophages (Fig. 4A-B), indicating a role of MerTK in prostate cancer cell efferocytosis specifically. MerTK inhibitory antibody Mer590 decreases total MerTK expression in M2 THP-1 macrophages (Fig. 4C). Mer590 decreases efferocytosis of LNCaP cells by M2 HMDMs 6 h following apoptotic LNCaP cell addition (Fig. 4D-F). In hi-myc prostate tumors aged to 2 months, *Mertk* KO mice have increased dead cells, suggesting that *Mertk* KO tumors have decreased prostate cancer efferocytosis in vivo (Fig. 4G).

### Mertk KO increases the anti-tumor macrophage phenotype and T cell infiltration in hi-myc prostate cancer tumors aged to 12 months

**Fig. 5:**
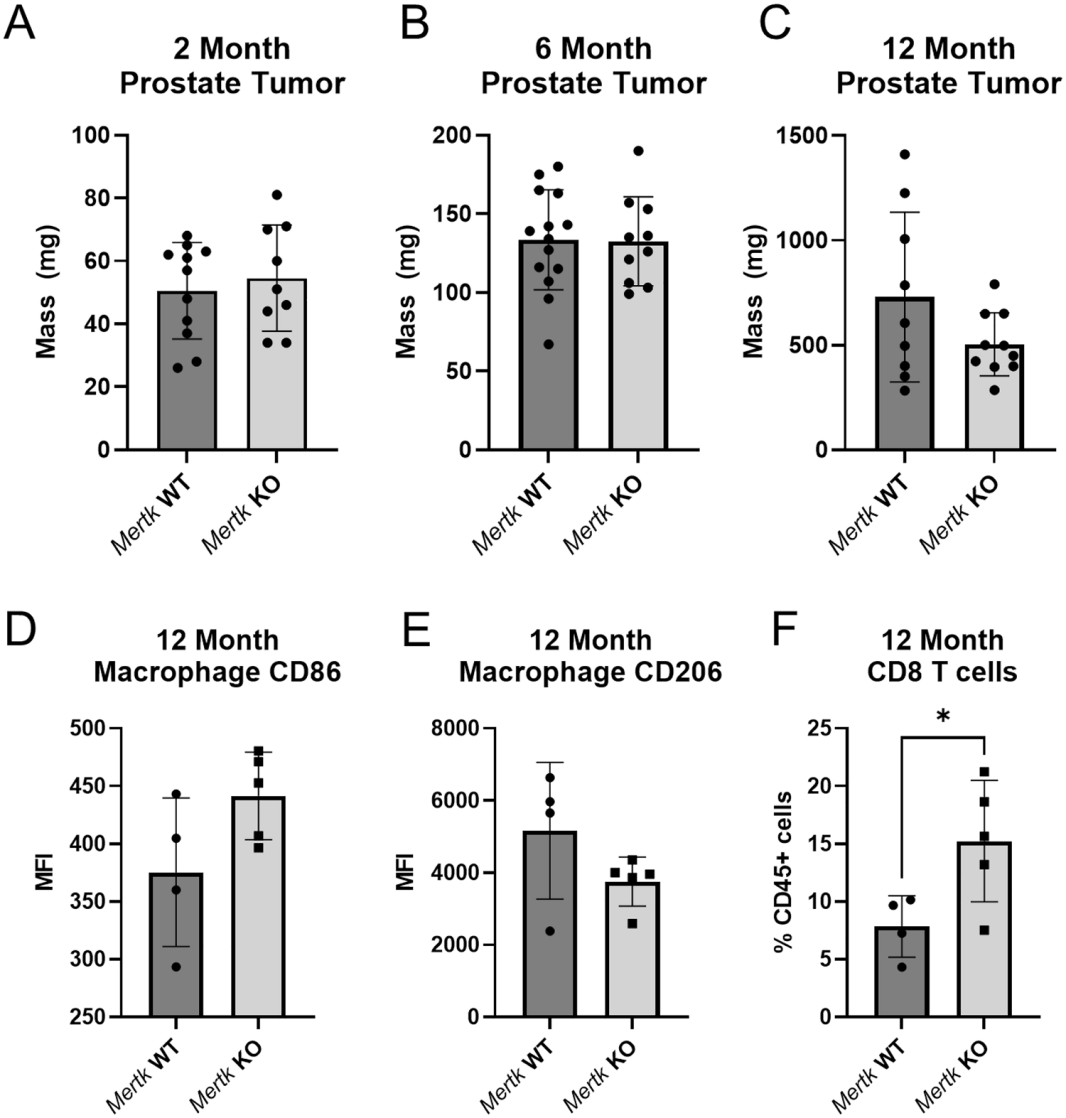
Immune cell composition in Mertk WT and KO hi-myc prostates. Ventral, dorsal, and lateral (VDL) prostate lobes in 2-month (**A**), 6-month (**B**), and 12-month (**C**) cohorts. Median fluorescence intensity (MFI) of macrophage CD86 (**D**) and CD206 (**E**) in the 12-month cohort. CD8 T cells as a percentage of CD45 + cells in the 12-month cohort (**F**). Significance of bar graphs was determined by t-test with * *p* < 0.05. Additional immune characteristics across the different cohorts are in Supplementary Fig. 3

In mice aged to 2 months, *Mertk* KO mice trend to have increased VDL tumor mass compared *Mertk* WT mice (Fig. 5A). In mice aged to 6 months, there is no difference in VDL tumor mass between *Mertk* WT and *Mertk* KO (Fig. 5B). In mice aged to 12 months, *Mertk* KO mice trend to have smaller tumors than *Mertk* WT mice (Fig. 5C). Macrophages in these mice have increased CD86 MFI and decreased CD206 MFI (Fig. 5D, E). Additionally, in mice aged to 12 months, *Mertk* KO tumors have increased CD8 T cell infiltration (Fig. 5G). Additional immune cell infiltration in the 2-, 6-, and 12-month cohorts is summarized in Supplementary Fig. 3.

## Discussion

Although efferocytosis has been studied in many models, there remain only a few studies of efferocytosis in prostate cancer. We adapted existing in vitro flow cytometry protocols onto our human macrophage models. We sought to measure the differences in prostate cancer cell efferocytosis using human M1 and M2 macrophage models. M2 HMDMs efferocytosed LNCaP cells at higher levels than M1 HMDMs (Fig. 1C-E). Approximately 80% of M2 HMDMs efferocytosed an LNCaP cell(s) compared to only 50% of M1 HMDMs. Efferocytosis was also quantified by CFSE delta MFI to analyze the mass of LNCaP cells efferocytosed per macrophage. M2 HMDMs had a higher CFSE delta MFI than M1 HMDMs. Compared to quantifying efferocytosis as a percentage of macrophages, there was much more biological variation in CFSE delta MFI between the three replicates. This reflects that donor to donor, there is biological variation in the average mass of LNCaP cells that M2 HMDMs efferocytosed, and there is less biological variation in efferocytosis as a percentage of macrophages.

Similar to the HMDM model, more M2 THP-1 macrophages efferocytosed LNCaP cells than M1 HMDMs when quantified as a percentage of macrophages (Fig. 1F, G). There was no significant difference in CFSE delta MFI between M1 and M2 THP-1 macrophages (Fig. 1H). This suggests that more M2 THP-1 macrophages are efferocytosing than M1 THP-1 macrophages, but there is no significant difference in the average LNCaP mass efferocytosed per macrophage. Differences in CFSE delta MFI significance between the two macrophage models may be due to differences between the two models such as PMA treatment and a shorter polarization in the THP-1 macrophage model and/or that THP-1 macrophages are derived from monocytic leukemia cell line.

It has been reported in the literature that macrophages have an increased M2-like phenotype following efferocytosis, contributing to the hypothesis that efferocytosis is a tumor-promoting function of macrophages. It is unknown how efferocytosis of human prostate cancer cells changes human macrophage phenotype. We assessed changes in expression of pro-tumor macrophage markers CD206 and PDL1 following efferocytosis of LNCaP cells. PDL1 is reported to be expressed on macrophages in the TME and contributes to suppressing the anti-tumor immune response by binding PD1 on T cells [29, 30]. We observed an increase in the expression of CD206 and PDL1 in M2 HMDMs incubated with apoptotic LNCaP (Fig. 2). These data support that following prostate cancer efferocytosis, M2 human macrophages have an increased pro-tumor phenotype and can further suppress the anti-tumor immune response and support cancer progression.

Given that M2 human macrophages efferocytosed prostate cancer cells at higher levels than M1s and that efferocytosis of prostate cancer cells supports the pro-tumor macrophage phenotype, we investigated targeting efferocytosis as a potential therapy for prostate cancer. Tyro3, Axl, and MerTK mediate efferocytosis by macrophages, but their role in prostate cancer cell efferocytosis is unknown. There are inconsistencies regarding Tyro3 and Axl expression in M1 and M2 macrophage models reported in the literature. We surveyed the expression of these receptors on M1 and M2 human macrophages. Tyro3 expression on M1 and M2 macrophages varied between biological replicate and at the protein or mRNA level (Supplementary Fig. 4A-F). Axl protein was not detected in M1 or M2 macrophages, but *AXL* mRNA was elevated in M1 macrophages (Supplementary Fig. 4G-L). MerTK was expressed higher in M2 than M1 macrophages in our models with every technique employed: flow cytometry, Western Blot, NanoString mRNA panel, and qRT-PCR. (Fig. 3). This was observed consistently in THP-1 macrophages and with multiple biological replicates of HMDMs. It has been shown that MerTK is expressed higher in M2 than in M1 macrophages across many models. Furthermore, there is strong evidence that MerTK has a larger role in efferocytosis by macrophages than Axl and Tyro3 [19]. When MerTK is activated by its ligands, the receptors dimerize, leading to phosphorylation and initiation of signaling cascades to promote efferocytosis. Phosphorylated MerTK increased in LNCaP-efferocytosing M2 macrophages, suggesting a role in prostate cancer efferocytosis (Fig. 4A-B). Additionally, high MerTK expression in M2s may support prostate cancer efferocytosis through a non-kinase function. We conclude that MerTK is the strongest candidate among the TAM receptors for blocking prostate cancer cell efferocytosis.

We explored targeting MerTK to block prostate cancer efferocytosis using a MerTK targeting antibody in vitro and an in vivo knockout mouse model. Mer590 is a MerTK targeting antibody that leads to receptor internalization and degradation [31]. We confirmed that Mer590 decreased cell surface levels of MerTK in M2 THP-1 macrophages (Fig. 4C). When M2 HMDMs are incubated with Mer590 prior to LNCaP cell addition, efferocytosis is decreased (Fig. 4D-F). These data support MerTK as a target to decrease prostate cancer cell efferocytosis.

We explored targeting MerTK in vivo in the prostate cancer genetically engineered mouse model hi-myc. These mice express the human *MYC* oncogene driven by a prostate-specific probasin and androgen-regulated promoter. In this model, male mice develop prostate intraepithelial neoplasia around 2 weeks and prostate cancer adenocarcinoma by 3–6 months [32]. A prostate cancer tumor is a complex and dynamic microenvironment containing many different cell types and components that can influence growth of the cancer cells. This model is highly advantageous for studying prostate cancer because the tumor develops in the tissue of origin, allowing for appropriate influence of the endogenous prostate cancer tumor microenvironment. Additionally, this model allows different stages of disease progression to be tracked by aging mice to multiple timepoints.

We sought to understand MerTK’s role in tumor growth and immune infiltration in the FVB hi-myc prostate cancer model. We have previously shown that there is high macrophage infiltration in this mouse model [33]. Based on our in vitro data, targeting MerTK on macrophages decreased prostate cancer efferocytosis. We hypothesized that *Mertk* KO macrophages in hi-myc prostate tumors would have decreased efferocytosis. Due to technical challenges of measuring efferocytosis in vivo, we examined the abundance of dead cells as an indirect measurement for efferocytosis. Tissues with low efferocytosis will have high dead cell accumulation. In *Mertk* KO hi-myc tumors, there are increased dead cells compared to *Mertk* WT hi-myc tumors (Fig. 4G). This supports that macrophages in *Mertk* KO hi-myc tumors have decreased efferocytosis. We hypothesized that decreased efferocytosis would promote an increase in anti-tumor immune infiltration such as M1-like macrophages and CD8 T cells.

There was no statistically significant difference in tumor mass between *Mertk* WT and *Mertk* KO mice in any of the cohorts, although *Mertk* KO mice aged to 12 months trended toward having smaller tumor masses than *Mertk* WT mice (Fig. 5A-C). We were interested in whether this difference in tumor mass was due to efferocytosis. Since prostates from *Mertk* KO mice had increased dead cell accumulation (Fig. 4G), we infer that macrophages in *Mertk* KO prostate tumors have decreased efferocytosis. In the 12-month cohort, macrophages from *Mertk* KO tumors had trends of increased CD86 expression and decreased CD206 expression. CD86 and CD206 are well characterized M1 and M2 macrophage markers, respectively. These data support that in *Mertk* KO mice, macrophages have an increased anti-tumor M1-like macrophage phenotype. Further phenotype analysis with additional macrophage cell surface markers are needed to characterize differences between *Mertk* WT and KO mice in hi-myc prostate tumors. In the 12-month cohort, *Mertk* KO tumors had increased CD8 T cell infiltration (Fig. 5F). Representative examples of CD86, CD206, and CD8 flow cytometry plots are in Supplementary Fig. 3 J–L. These data support targeting MerTK-mediated efferocytosis to increase anti-tumor immune infiltrate, including CD8 T cells and M1-like macrophages.

Given that MerTK only had a minor role in prostate cancer efferocytosis in vitro (Fig. 4D-F), targeting macrophage MerTK may not be sufficient to inhibit prostate cancer growth. Macrophages in *Mertk* KO hi-myc prostates may only have partially decreased efferocytosis, resulting in no difference in tumor mass or macrophage phenotype in early stages of the model. These data suggest that at later stages of the hi-myc model, *Mertk* KO was sufficient to increase CD8 T cells and trend toward lower tumor mass (Fig. 5C, F). Future studies will investigate targeting all three TAM receptors to further block efferocytosis of prostate cancer cells. Additionally, TAM receptor inhibition combined with cytotoxic therapy or radiotherapy may serve as a stronger strategy to inhibit prostate cancer growth by synergizing the induction of apoptosis in tumor cells with blocking of efferocytosis by macrophages.

## Conclusions

Efferocytosis is an emerging area of interest in cancer biology, although there are limited studies of efferocytosis in prostate cancer. Both M2 HMDMs and M2 THP-1 macrophages engaged in higher levels of LNCaP efferocytosis than their respective M1 counterparts. Following LNCaP efferocytosis, M2 HMDMs had an increased pro-tumor phenotype. This work supports efferocytosis as a tumor-promoting function of macrophages and suggests targeting efferocytosis may provide a novel prostate cancer therapy. Inhibiting MerTK decreased LNCaP cell efferocytosis in our in vitro efferocytosis assays. In the hi-myc prostate cancer mouse model, macrophage cell surface marker analysis indicated that *Mertk* KO mice had an increased M1-like anti-tumor phenotype. Additionally, *Mertk* KO mice had increased CD8 T cell infiltration. These data support MerTK as a target for blocking efferocytosis as a novel therapy for prostate cancer.

### Supplementary Information

Below is the link to the electronic supplementary material.

Supplementary file1 (DOCX 1819 kb)

## Data Availability

The datasets generated during and/or analyzed during the current study are available from the corresponding author on reasonable request.
