# Supplementary material for: Targeting MerTK decreases efferocytosis and increases anti-tumor immune infiltrate in prostate cancer
Source: Med Oncol. 2023 Aug 29;40(10):284. doi: 10.1007/s12032-023-02153-z (PMC10465384; doi:10.1007/s12032-023-02153-z)
Supplement: Supplementary file 1 — Supplementary file1 (DOCX 1819 kb) [file 12032_2023_2153_MOESM1_ESM.docx]

| **Target** | **Target Antibody Catalog Number** | **Isotype** | **Isotype Antibody Catalog Number** | **Fluorophore** |
| --- | --- | --- | --- | --- |
| Axl | FAB154P  R&D Systems | Mouse IgG1 | IC002P  R&D Systems | PE |
| CD206 | 130-100-085, Miltenyi Biotec | Mouse IgG1 | 130-113-199, Miltenyi Biotech | FITC |
| PDL1 | 329724, BioLegend | Mouse IgG2b, κ | 400350, BioLegend | BV605 |
| MerTK | FAB8912P  R&D Systems | Mouse IgG2B | IC0041P  R&D Systems | PE |
| Tyro3 | FAB859P R&D Systems | Mouse IgG1 | IC002P  R&D Systems | PE |

**Supplementary Table 1.** Macrophage cell surface marker flow cytometry antibodies.

| **Gene** | **Forward Primer** | **Reverse Primer** |
| --- | --- | --- |
| *TYRO3* | 5’-CAGCCGGTGAAGCTCAACT-3’ | 5’-TGGCACACCTTCTACCGTGA-3’ |
| *AXL* | 5’-CTCAGAATCACCTCCCTGCA-3’ | 5’-AGCATCCTGGAGCCAGAGTA-3’ |
| *MERTK* | 5’-ACCTCTGTCGAATCAAAGCCC-3’ | 5’-CTGCACACTGGTTATGCTGAA-3’ |
| *UBC* | 5’-ATTTGGGTCGCGGTTCTTG-3’ | 5’-TGCCTTGACATTCTCGATGGT-3’ |
| *RPL13A* | 5’-CCTGGAGGAGAAGAGGAAAGAGA-3’ | 5’-TTGAGGACCTCTGTGTATTTGTCAA-3’ |

**Supplementary Table 2.** **Forward and reverse primer sequences for TAM receptor gene expression and housekeeping genes.**

| Target | Target antibody clone | Target antibody catalog number | Isotype | Isotype antibody clone | Isotype antibody catalog number | Fluoro-phore | Concen-tration | Company |
| --- | --- | --- | --- | --- | --- | --- | --- | --- |
| Extracellular | | | | | | | | |
| F4/80 | T45-2342 | 746070 | IgG2a,κ | R35-95 | 566413 | BB700 | 0.125ug | BD Biosciences |
| CD11b | M1/70 | 561039 | IgG2b,κ | A95-1 | 552773 | APCCy7 | 0.005ug | BD Biosciences |
| CD45 | 30-F11 | 561487 | IgG2b,κ | R35-38 | 562951 | PECy7 | 0.125ug | BD Biosciences |
| CD86 | GL-1 | 105032 | IgG2a,κ | RTK2758 | 4000536 | BV421 | 0.125ug | Bio Legend |
| Intracellular | | | | | | | | |
| CD206 | MR5D3 | 565250 | IgG2a,κ | R35-95 | 557690 | AF647 | 0.125ug | BD Biosciences |
| Live/Dead: LIVE/DEAD Fixable Yellow, 1uL/mL (L34959, Thermo Fisher Scientific) | | | | | | | | |

**Supplementary Table 3.** **Myeloid flow cytometry antibody panel.**

| Target | Target antibody clone | Target antibody catalog # | Isotype | Isotype antibody clone | Isotype antibody catalog # | Fluoro-phore | Concen-tration | Company |
| --- | --- | --- | --- | --- | --- | --- | --- | --- |
| Extracellular | | | | | | | | |
| CD49b | DX5 | 562453 | IgM,κ | R4-22 | 562489 | PECF594 | 0.125ug | BD Biosciences |
| CD3 | 17A2 | 557869 | IgG2b,κ | A95-1 | 557691 | AF647 | 0.125ug | BD Biosciences |
| CD4 | RM4-5 | 557956 | IgG2a,κ | R35-95 | 557963 | AF700 | 0.125ug | BD Biosciences |
| CD8 | 53-6.7 | 560182 | IgG2a,κ | R35-95 | 560197 | APCH7 | 0.125ug | BD Biosciences |
| CD45 | 30-F11 | 561487 | IgG2b,κ | R35-38 | 562951 | BV510 | 0.125ug | BD Biosciences |
| Live/Dead: FVS570, 1uL/mL (564995, BD Biosciences) | | | | | | | | |

**Supplementary Table** **4. Lymphocyte flow cytometry panel.**

| **Cell type** | **Marker definition/gating strategy** |
| --- | --- |
| Macrophages | LIVE/DEAD Fixable Yellow^-^ CD45^+^ CD11b^+^ F4/80^+^ |
| CD4 helper T cells | FVS570^-^ CD45^+^CD3^+^CD4^+^ |
| CD8 cytotoxic T cells | FVS570^-^ CD45^+^CD3^+^CD8^+^ |
| NK cells | FVS570^-^ CD45^+^CD49b^+^ |

**Supplementary Table** **5. Flow cytometry immune cell markers.**


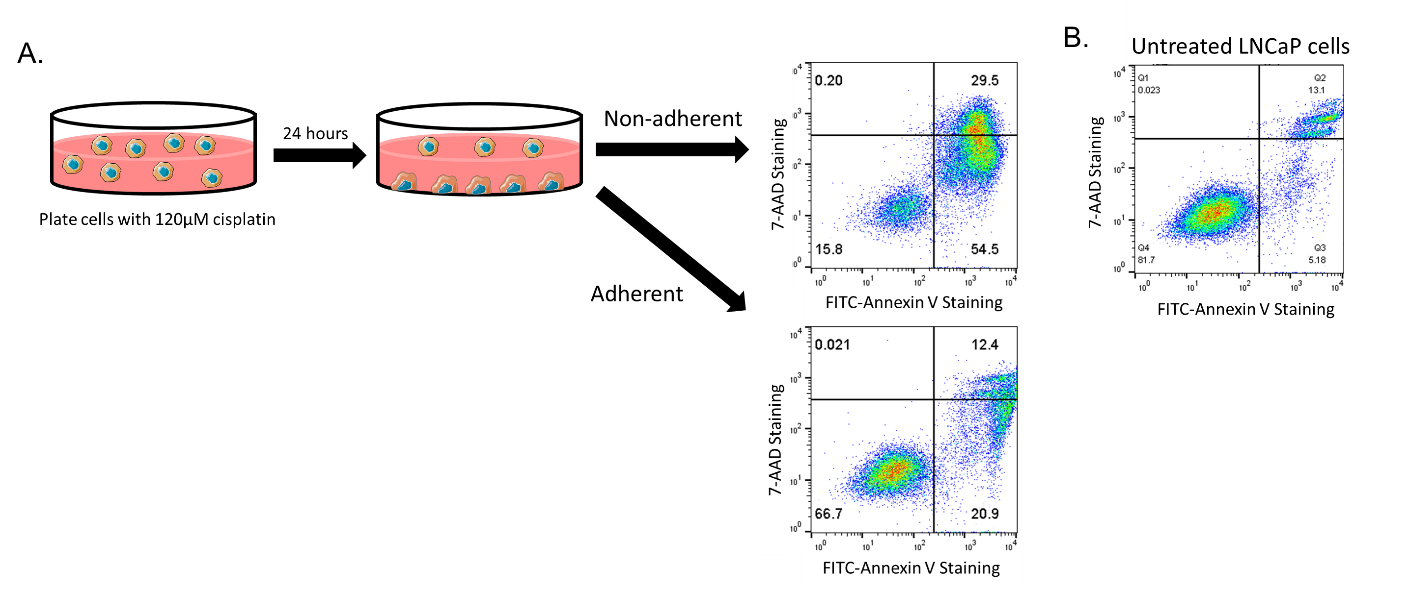


**Supplementary Figure 1. LNCaP apoptosis with 1 day chemotherapy incubation.** LNCaP cells were plated with 120μM cisplatin in suspension. After a 24 hour incubation, FITC-Annexin V and 7-AAD apoptosis staining on non-adherent and adherent cells (A). As a control, untreated LNCaP cells showed low apoptosis (B). Quadrant gating was defined using unstained and single stain controls.

**
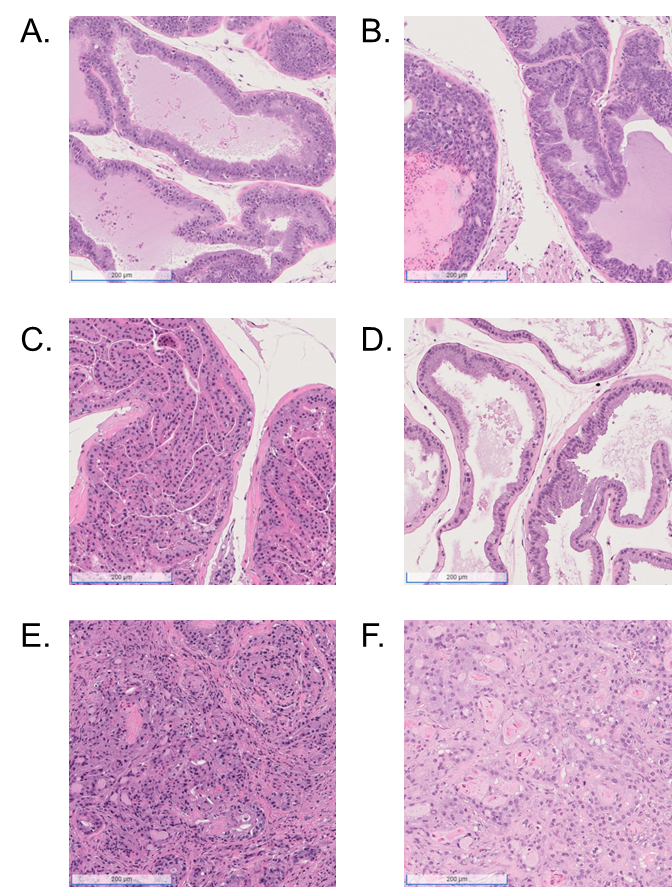
Supplementary Figure 2. Representative histology images of *Mertk* WT and *Mertk* KO hi-myc prostate tumors.** *Mertk* WT aged to 2 months (A), *Mertk* KO aged to 2 months (B), *Mertk* WT aged to 6 months (C), *Mertk* KO aged to 6 months (D), *Mertk* WT aged to 12 months (E), and *Mertk* KO aged to 12 months (F).


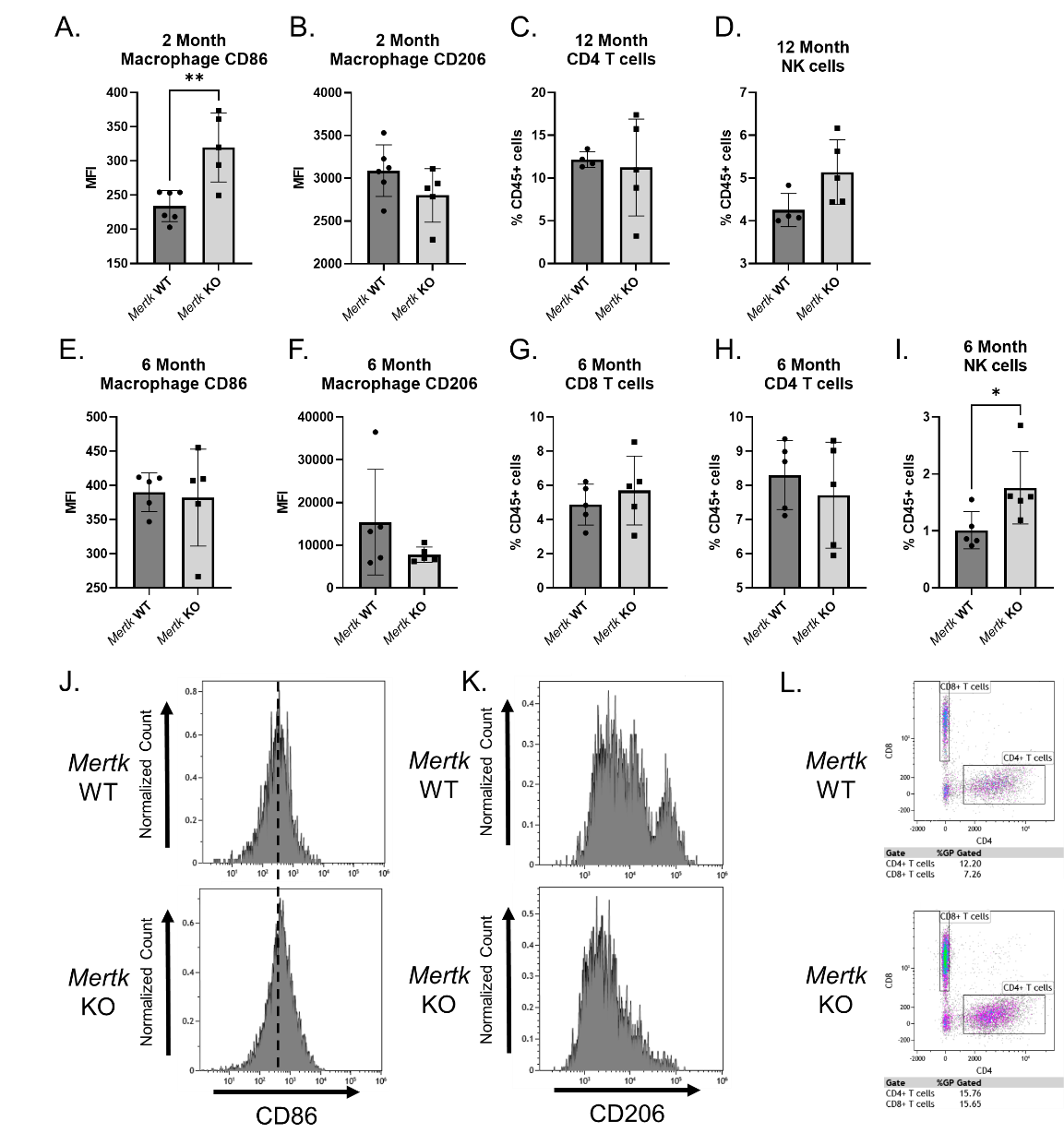


**Supplementary Figure 3. Additional immune cell composition in hi-myc prostates.** Macrophage CD86 (A) and CD206 (B) MFI in the 2-month cohort. CD4 T cells (C) and NK cells (D) as a percentage of CD45+ cells in the 12-month cohort. CD86 (E) and CD206 (F) MFI in the 6-month cohort. CD8 T cells (G) CD4 T cells (H) and NK cells (I) as a percentage of CD45+ cells in the 6-month cohort. Representative flow plots of CD86 (J), CD206 (K) and CD8/CD4 (L). %GP Gated indicated % Grandparent gated (%CD45). Significance of bar graphs were determined by t test with * p < 0.05 and ** p < 0.01.


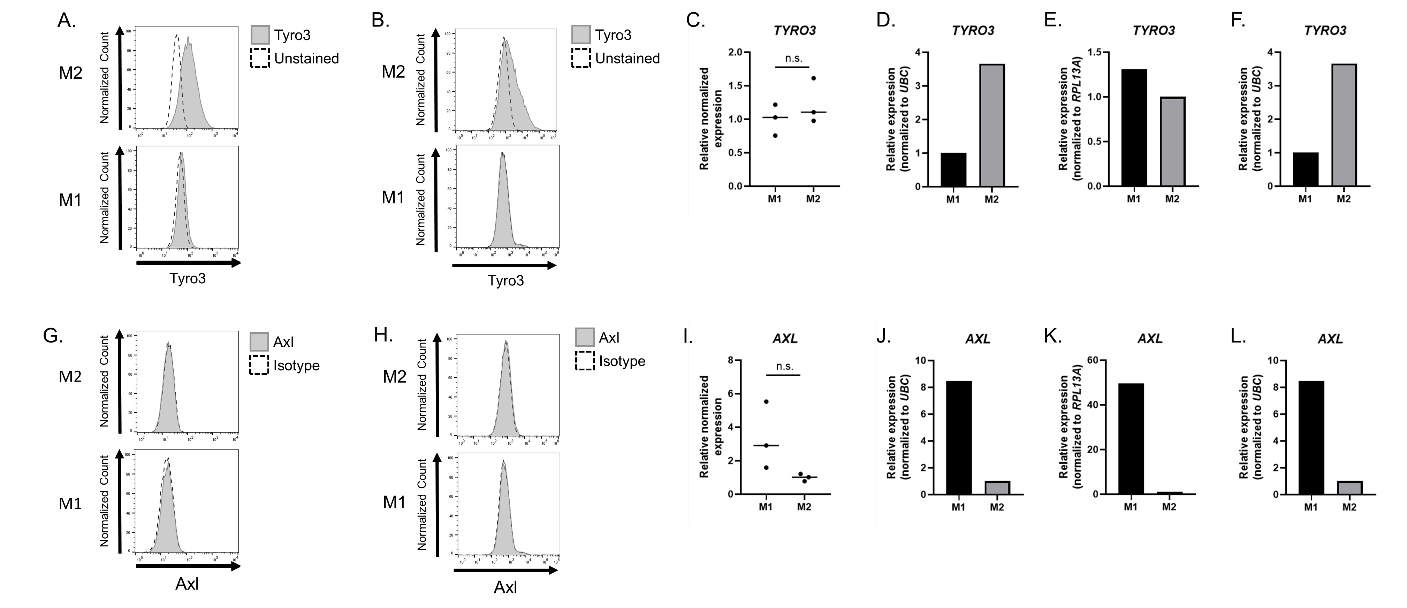
**Supplementary Figure 4. Tyro3 and Axl expression in M1 and M2 human macrophages.** Flow cytometry analysis of Tyro3 on M1 and M2 HMDMs (A) and THP-1 macrophages (B). *TYRO3* mRNA expression by NanoString analysis in three biological replicates of M1 and M2 HMDMs (C). *TYRO3* mRNA expression by qRT-PCR in two biological replicates of M1 and M2 HMDMs (D, E) and M1 and M2 THP-1 macrophages (F). Tyro3 flow cytometry data in HMDMs is representative of two of three biological replicates. Flow cytometry analysis of Axl on M1 and M2 HMDMs (G) and THP-1 macrophages (H). *AXL* mRNA expression by NanoString analysis in three biological replicates of M1 and M2 HMDMs (I). *AXL* mRNA expression by qRT-PCR in two biological replicates of M1 and M2 HMDMs (J, K) and M1 and M2 THP-1 macrophages (L). Axl flow cytometry data in HMDMs is representative of three biological replicates. ns = not significant.
